# Regulation of Integrin Subunit Alpha 2 by miR-135b-5p Modulates Chemoresistance in Gastric Cancer

**DOI:** 10.3389/fonc.2020.00308

**Published:** 2020-03-13

**Authors:** Qi Wang, Tianyu Cao, Kai Guo, Yao Zhou, Hao Liu, Yanan Pan, Qiuqiu Hou, Yongzhan Nie, Daiming Fan, Yuanyuan Lu, Xiaodi Zhao

**Affiliations:** ^1^State Key Laboratory of Cancer Biology, National Clinical Research Center for Digestive Diseases and Xijing Hospital of Digestive Diseases, Xijing Hospital, Fourth Military Medical University, Xi'an, China; ^2^Department of Burns and Cutaneous Surgery, Xijing Hospital, Fourth Military Medical University, Xi'an, China; ^3^Guangxi Key Laboratory of Biological Targeting Diagnosis and Therapy Research, Collaborative Innovation Center for Targeting Tumor Diagnosis and Therapy, Guangxi Medical University, Nanning, China; ^4^College of Life Sciences, Northwest University, Xi'an, China; ^5^National Institute of Biological Sciences, Beijing, China

**Keywords:** ITGA2, miR-135b-5p, gastric cancer, chemoresistance, MAPK pathway, EMT

## Abstract

Chemotherapy has substantially improved gastric cancer (GC) patient outcomes in the past decades. However, the development of chemotherapy resistance has become the major cause of treatment failure. Although numerous molecules have been implicated in GC chemoresistance, its pathological mechanisms are still unclear. Here, we found that integrin subunit alpha 2 (ITGA2) is upregulated in chemoresistant GC cells and that increased ITGA2 levels correlated with the poor prognosis of GC patients who received chemotherapy. ITGA2 overexpression led to elevated chemotherapy resistance and drug-induced apoptosis inhibition in GC cells. ITGA2 knockdown resulted in restored chemosensitivity and increased apoptosis in chemoresistant GC cells both *in vitro* and *in vivo*. NanoString analysis revealed a unique signature of deregulated pathway expression in GC cells after ITGA2 silencing. The MAPK/ERK pathway and epithelial-mesenchymal transition (EMT) were found to be downregulated after ITGA2 knockdown. miR-135b-5p was identified as a direct upstream regulator of ITGA2. miR-135b-5p overexpression reduced chemoresistance and induced apoptosis in GC cells and attenuated ITGA2-induced chemoresistance and antiapoptotic effects by inhibiting MAPK signaling and EMT. In conclusion, this study underscored the role and mechanism of ITGA2 in GC and suggested the novel miR-135b-5p/ITGA2 axis as an epigenetic cause of chemoresistance with diagnostic and therapeutic implications.

## Introduction

Gastric cancer (GC) is the third leading cause of cancer-related deaths worldwide and is responsible for over a million new cases and estimated 783,000 deaths globally in 2018 (1). Chemotherapy is the recommended treatment for unresectable or recurrent GC (2). The effectiveness of chemotherapy largely depends on the resistance to chemotherapy, and chemoresistance has therefore become the main cause of treatment failure (3). Based on previous studies, many factors are associated with the development of chemoresistance, including changes in the activity of membrane transporters, increased drug metabolism, alteration of drug targets, epithelial-mesenchymal transition (EMT), and tumor heterogeneity, all of which can affect the sensitivity of cancer cells to chemotherapeutic drugs (4, 5). Furthermore, drug resistance-related genes (such as MDR and LRP) and various signaling pathways (such as MAPK, Wnt, and Notch) are reported to be significant causes of chemoresistance (6). However, the molecular mechanisms of chemoresistance are still not fully understood, and more research is needed to discover and develop effective biomarkers and targets for GC chemoresistance.

Integrins are cell surface receptors and play multifaceted roles as signaling molecules, mechanotransducers and key components of the cell migration machinery (7). They are transmembrane αβ heterodimers and include at least 18 α and 8 β subunits in humans (8). In cancer, integrins trigger and are involved in the regulation of diverse cellular functions crucial to tumor initiation, progression, and metastasis (9). More recently, integrins have been reported to be involved in drug resistance, which may be due to the selection for tumor cells already expressing certain integrins or to the regulation of integrin gene expression (10). For instance, increased integrin subunit alpha 1 (ITGA1) actuated gemcitabine resistance by cooperating with TGF-β in pancreatic cancer (11). Depletion of integrin subunit beta 1 (ITGB1) enhanced the sensitivity of tumor cells to docetaxel in esophageal squamous cell carcinoma (12). In addition, integrin subunit beta 3 (ITGB3) was identified as a target to overcome chemoresistance in mesenchymal lung cancer, and inhibition of ITGB3 sensitizes cancer cells to chemotherapy by regulating the NF-κB pathway (13). Integrin subunit alpha 2 (ITGA2) encodes a member of the integrin α chain family, which forms a heterodimer with the β1 subunit and regulates the adhesion of platelets and other kinds of cells to the extracellular matrix (ECM) (14, 15). Recently, studies showed that aberrant expression of ITGA2 was associated with metastatic behavior in breast cancer, liver cancer, and colorectal cancer (16–19). In GC, blocking ITGA2 with specific antibodies was reported to inhibit cell migration and induce apoptosis (20). However, the specific role and underlying mechanisms of ITGA2 in GC chemoresistance are largely unknown.

The initiation and progression of cancer is thought to be driven by combinations of genetic and epigenetic alterations (21). MicroRNAs (miRNAs) are non-coding RNAs of 18–24 nucleotides in length and directly modulate gene expression at the posttranscriptional level by binding to the 3′-untranslated region (3′-UTR) of target mRNAs (22). Genome-wide analysis has demonstrated that miRNA expression is dysregulated in most cancer types, which may contribute to dysregulate critical genes involved in the development and evolution of cancer (23). More recently, a variety of mechanisms have been postulated for the roles that miRNAs play in resistance to cancer treatments, and miRNA-based gene therapy may provide a novel approach for drug resistance (24–26). For example, miR-27a was decreased in bladder cancer and restored miR-27a re-sensitized cisplatin resistance by targeting the cystine/glutamate exchanger SLC7A11 (27). miR-340-5p was reduced in breast cancer, and its overexpression inhibited drug resistance to docetaxel by targeting LRG5 via the Wnt/β-catenin pathway (28, 29). We previously found that low expression of miR-15b and miR-16 was detected in drug-resistant GC cells, which medicated sensitivity to vincristine (VCR) by directly regulating Bcl-2 (30). In addition, we found that miR-100 and miR-125b were often overexpressed and participated in cetuximab resistance through the Wnt/beta-catenin signaling pathway in colorectal cancer (31). However, whether ITGA2 is regulated by miRNA in GC chemoresistance remains to be elucidated.

In this study, we found that ITGA2 is often increased in GC cells and tissues, especially in chemoresistant GC cells. The upregulation of ITGA2 correlated with the poor prognosis of GC patients who received chemotherapy. ITGA2 silencing induced apoptosis and restored GC cell chemotherapy responsiveness both *in vitro* and *in vivo*. Mechanistically, repression of ITGA2 inhibited the MAPK/ERK pathway as well as the EMT process. miR-135b-5p was identified as a direct regulator of ITGA2, and the restoration of miR-135b-5p resulted in anti-chemoresistance effects, which were similar to the effects of ITGA2 inhibition. Our results showed a pro-chemoresistance effect of ITGA2 and identified its miRNA regulatory mechanism. Identification of this novel miR-135b-5p/ITGA2 axis sheds new light on the understanding of chemoresistance in GC and may provide therapeutic targets for GC treatment.

## Materials and Methods

### Cell Culture and Treatment

Human normal gastric mucosal cell lines GES-1 and GC cell lines AGS, MKN45, MKN28, SNU1, SNU16, BGC823, and SGC7901 were preserved in the State Key Laboratory of Cancer Biology (CBSKL) inventory. All cell lines were authenticated by cellular morphology and short tandem repeat analysis. The multidrug resistant cell lines SGC7901/VCR and SGC7901/ADR were screened stepwise with VCR and ADR in our laboratory previously (32). All cells were cultured in Dulbecco's modified Eagle's medium (DMEM, Gibco, USA) supplemented with 10% fetal bovine serum (FBS, Gibco, USA) and 100 U/mL penicillin and 100 mg/mL streptomycin at a 37°C humidified incubator with 5% CO_2_. To maintain the MDR phenotype, VCR and ADR (MCE, USA) were added at final concentrations of 1 μg/ml and 0.5 μg/ml to the culture media of SGC7901/VCR and SGC7901/ADR cells.

### Quantitative Real-Time Polymerase Chain Reaction (qRT-PCR)

Total RNA was extracted using the RNeasy Mini Kit (Qiagen GmbH, Germany) and reverse transcribed into cDNA using the Prime Script RT Reagent Kit (TaKaRa, Japan). The RT-PCR primers for miR-135b-5p, miR-181b-5p, and U6 were purchased from RiboBio (China). The PCR primers for ITGA2 were 5′-GAGAACAACAGGTGACTT-3′ (forward) and 5′-CTCTCCTGTATGATGCTG-3′ (reverse). The PCR primers for GAPDH were 5′-AGAAGGCTGGGGCTCATTTG-3′ (forward) and 5′-GAAGACTGTGGATGGCCCCT-3′ (reverse). Real-time quantitative PCR assays were performed with SYBR Premix Ex Taq II (TaKaRa, Japan) at 95°C for 30 s, followed by 39 cycles of 95°C for 5 s and 60°C for 30 s. The expression levels of GAPDH and U6 were used as internal controls.

### Western Blotting Analysis

Total proteins are extracted by RIPA lysis (Beyotime, China) containing protease and phosphatase inhibitors. The BCA Protein Assay Kit (Thermo, USA) was used to detect the protein concentration. Thirty micrograms of denatured protein were separated via SDS-PAGE followed by transfer to nitrocellulose filter membranes (Millipore, USA). The protein bands were exposed in a Bio-Rad Imaging System (Bio-Rad, USA) after incubation with the primary and secondary antibodies. The antibodies used were against ITGA2 (Abcam, UK, #133557), β-actin (Cell Signaling Technology, USA, #4970), ERK (Cell Signaling Technology, USA, #4695), p-ERK (Cell Signaling Technology, USA, #4370), MEK (Cell Signaling Technology, USA, #8727), p-MEK (Cell Signaling Technology, USA, #9154), MDR1 (Cell Signaling Technology, USA, #12683), E-cadherin (Proteintech, USA, 20874-1-AP), N-cadherin (Cell Signaling Technology, USA, #13116), Vimentin (Cell Signaling Technology, USA, #5741), PCNA (Cell Signaling Technology, USA, #13110), Bax (Cell Signaling Technology, USA, #5023), and Bcl-2 (Cell Signaling Technology, USA, #4223). Original images of blots are presented in Figure S3.

### Constructs, Oligonucleotides, Infection, and Transfection

ITGA2 lentiviral vectors (overexpression and shRNA) were purchased from GeneChem (China). Target cells were transfected with 1 × 10^7^ lentivirus-transducing units using polybrene as recommended. Empty lentiviral vectors were also transfected as a negative control. Puromycin (MCE, China) was employed to screen cells with antibiotic labels. The cells were collected for future study. The pENTER-ITGA2-expressing plasmid was obtained from Addgene (USA), and its 3′-UTR was subcloned between the *MluI* and *XhoI* sites. The miR-135b-5p mimic and miRNA mimic negative control were chemically synthesized and purified by RiboBio (China). We transfected miRNA mimics and plasmids with Transfect-mate (GenePharma, China) according to the manufacturer's recommendations.

### Cell Proliferation and IC50 Assay

For the IC50 assay, 4,000 cells were seeded into a well of a 96-well plate. The culture medium was changed, and 5-FU was added by multiple proportion dilution 12 h later. Then, 60 h later, CCK-8 (DOJINDO, Japan) was added according to the manufacturer's protocol, followed by incubation at 37°C for 2 h. The absorbance was read by a chemiluminescence measuring instrument (Bio-Rad, USA). For the cell proliferation assay, 3,000 cells were seeded, and the absorbance values were measured every 24 h for a total of 4 times.

### Cell Viability Analysis

The LIVE/DEAD viability/cytotoxicity kit (Thermo, USA) was used to perform cell viability analysis. Cells were seeded into 24-well plates, followed by chemotherapy drug treatment on the second day. Cell samples were stained following the directions on the fourth day and analyzed with a fluorescence microscope.

### *In vivo* Drug Resistance Assay

A total of 5 × 10^6^ SGC7901/ADR-shITGA2 cells or negative control cells were inoculated subcutaneously into two sides of the thigh in nude mice (obtained from the Fourth Military Medical University Animal Care). The ADR and 5-FU treatments were given intraperitoneally three times a week after the inoculations. These mice were sacrificed 3 weeks later, and the subcutaneous tumor tissues were removed; the tissues were then fixed, embedded, and sliced. All animal studies complied with the Fourth Military Medical University animal use guidelines, and the protocol was approved by the Fourth Military Medical University Animal Care Committee.

### Tissue Microarrays and Immunohistochemistry

The tissue microarrays were ST722, ST1004a (Alenabio, China), and HStmA050Me01 (Outdo Biotech, China). The tissue microarray staining was performed with an anti-ITGA2 antibody according to the instructions of the immunohistochemical kit (ZSGB-BIO, China). The tissues from subcutaneous tumors were stained with anti-Ki67 (Abcam, UK, #15580) and anti-Cleaved Caspase-3 (Cell Signaling Technology, USA, #9661) antibodies. Immunohistochemical (IHC) results were graded according to staining intensity and proportion of positive cells. Staining intensity was divided into 4 grades: 0, negative; 1, weak; 2, moderate; and 3, strong. Staining proportion included 0, <1%; 1, 1–25%; 2, 26–50%; 3, 51–75%; and 4, 76–100%. IHC scores, equaling the proportion of staining intensity times, were divided into negative (–, score: 0), weak (+, score: 1–4), moderate (++, score: 5–8), and strong (+++, score: 9–12). Negative and weak are considered low expression, while moderate and strong are considered high expression.

### NanoString PanCancer Pathways Analysis

For signaling pathway analysis, mRNA extracted from cell lysates was treated with nCounter Human PanCancer Pathways Panel (NanoString, USA). We used 100 ng of total RNA as an input for the sample preparation according to the manufacturer's recommendations. Samples and probes were hybridized overnight at 65°C, processed automatically at the pretreatment station, and transmitted to the digital analyzer for high-density scanning for data collection. Normalization and pairwise comparisons were performed using NanoString nSolver software. The data were analyzed by nSolver 4.0 software (NanoString, USA).

### Luciferase Reporter Assay

Wild-type and mutant ITGA2 promoter sequences were obtained by PCR amplification and connected to the psiCHECK-2 vectors. Plasmids with the wild-type ITGA2 promoter and mutant ITGA2 promoter were cotransfected with miR-135b-5p into 293T cells. The Dual-luciferase Reporter System Kit (Promega, USA) was employed to detect the luciferase activity, which was calculated with the following formula: relative activity = sample activity/control activity. GloMax^TM^ 20/20 (Promega, USA) was used for data analysis.

### Statistical Analysis

All analyses were performed using SPSS 22.0 (SPSS, USA). Statistical significance was assessed by Student's *t*-test, ANOVA, or χ^2^ tests. *P* < 0.05 were considered statistically significant, and all data are presented as the mean± standard deviation (SD).

## Results

### ITGA2 Is Upregulated in GC Cells and Tissues

**Figure 1:**
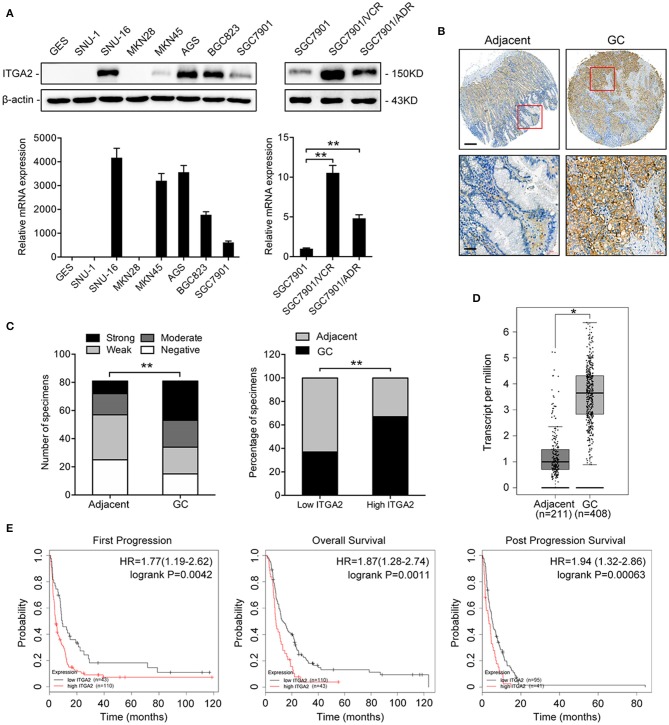
ITGA2 expression in GC cell lines, GC tissues and adjacent normal tissues. **(A)** Western blotting analysis and qRT-PCR analysis of ITGA2 expression in the GES and GC cell lines SUN-1, SUN-16, MKN28, MKN45, AGS, BGC823, SGC7901, SGC7901/VCR, and SGC7901/ADR. **(B,C)** ITGA2 expression in GC tissues was elevated compared to that in adjacent normal tissues by immunohistochemistry analysis. **(D,E)** Survival databases revealed that ITGA2 was increased in GC tissues and was related to poor prognosis after 5-FU treatment. Data represent mean ± SD, from three replicates. *P < 0.05, **P < 0.01.

To determine the expression pattern of ITGA2 in GC, we detected the ITGA2 protein and mRNA levels in an immortalized gastric epithelial cell line GES and in a panel of GC cell lines. Compared to that in GES cells, ITGA2 expression was significantly elevated in 5 out of 7 GC cells at both the protein and mRNA levels. We further found that ITGA2 was distinctly increased in SGC7901/VCR and SGC7901/ADR cells, which are chemoresistant GC cells derived from SGC7901 by stepwise screening with chemotherapy drug VCR and Adriamycin (ADR), respectively (Figure 1A) (32). Furthermore, a tissue microarray containing 81 pairs of GC tissues and matched adjacent normal tissues was used to detect the ITGA2 levels, and the results showed that ITGA2, mainly located in the cytoplasm and plasma membrane, was overexpressed in GC tissues compared to its expression in adjacent tissues (Figures 1B,C). Data from the GEPIA database (33) also show that ITGA2 expression was higher in GC tissues than in normal tissues (Figure 1D). Importantly, ITGA2 was correlated with the prognosis of GC patients who received 5-fluorouracil (5-FU) treatment, and an increased level of ITGA2 was correlated with decreased overall survival, first progression and post-progression survival times according to the Kaplan-Meier Plotter database (34) (Figure 1E). Taken together, these results showed that ITGA2 is upregulated in GC cells and tissues, especially in chemoresistant GC cells, suggesting its promoting effects on the chemoresistance of GC.

### ITGA2 Regulates the Chemosensitivity of GC Cells to Chemotherapeutic Drugs

**Figure 2:**
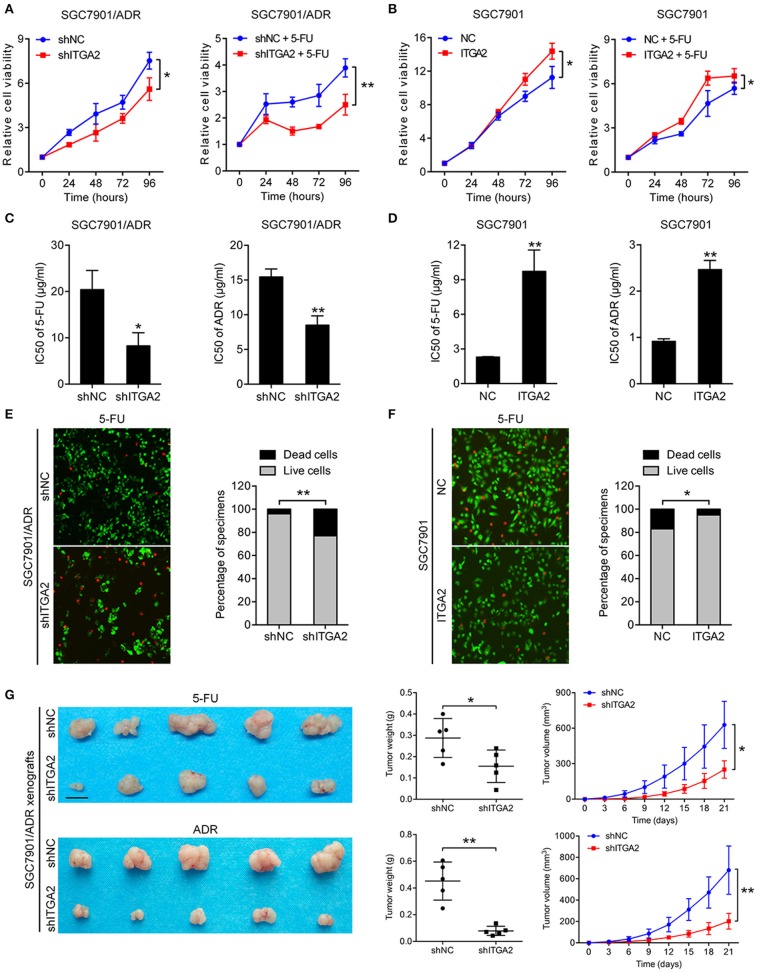
ITGA2 regulated chemoresistance in SGC7901 and SGC7901/ADR cells. **(A)** Cell proliferation analysis of SGC7901 cells when treated without or with 5-FU. **(B)** Cell proliferation analysis of SGC7901/ADR cells when treated without or with 5-FU. **(C,D)** IC50 of ADR and 5-FU when ITGA2 was overexpressed in SGC7901 cells and inhibited in SGC7901/ADR cells. **(E,F)** LIVE/DEAD viability analysis in response to 5-FU when overexpressed ITGA2 in SGC7901 cells and inhibited ITGA2 expression in SGC7901/ADR cells. **(G)** SGC7901/ADR cells with shITGA2 or vector control were injected into nude mice. Photos of xenograft tumors are shown on the right. Tumor weight and tumor growth curves are shown on the left. Data represent mean ± SD, from three replicates. *P < 0.05, **P < 0.01.

To investigate the role of ITGA2 in GC chemoresistance, we established gain- and loss-of-function cell models by infecting GC cells with a lentivirus expressing ITGA2 or silencing ITGA2 (shITGA2) (Figure S1A). CCK-8 assays showed that cell proliferation was inhibited in SGC7901/ADR-shITGA2 cells, and this inhibitory effect was enhanced when treated with 5-FU (Figure 2A). Overexpression of ITGA2 in the parental SGC7901 cells strengthened the proliferation ability in the presence or absence of 5-FU treatment (Figure 2B). Furthermore, IC50 assays were employed to determine chemoresistance in GC cells, and the results showed that the IC50 of 5-FU and ADR was remarkably decreased in SGC7901/ADR-shITGA2 cells but significantly increased when ITGA2 was overexpressed in SGC7901 cells (Figures 2C,D). In addition, LIVE/DEAD viability analysis showed that downregulation of ITGA2 increased, and upregulation of ITGA2 reduced, 5-FU- and ADR-induced apoptosis (Figures 2E, F, Figure S1B). To further determine whether ITGA2 confers chemoresistance *in vivo*, SGC7901/ADR-shITGA2 or control cells were injected subcutaneously into each flank of nude mice. Then, 5-FU or ADR was injected intraperitoneally after the tumor volume reached approximately 100 mm^3^. Tumor size and weight were significantly reduced by 5-FU or ADR treatment in mice implanted with ITGA2-silenced GC cells (Figure 2G). Immunohistochemical (IHC) staining showed that xenografts from the ITGA2-silenced group presented lower Ki-67 staining and higher Cleaved Caspase-3 staining than those from the control group upon 5-FU or ADR treatment (Figure S1C). Taken together, these results indicate that ITGA2 promoted the chemoresistance of GC cells both *in vitro* and *in vivo*.

### ITGA2 Activates the MAPK Pathway and Induces EMT in GC Cells

**Figure 3:**
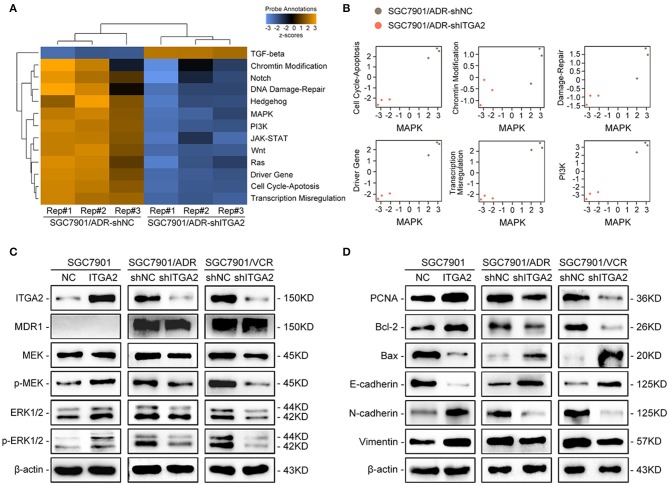
The effect of ITGA2 on the MAPK pathway in GC cells. **(A,B)** Heatmap and pathway panel plots summarizing the changed pathways when ITGA2 was inhibited in SGC7901/ADR cells. **(C)** Protein levels of MDR1 and the MAPK pathway markers were measured in SGC7901 cells with ITGA2 overexpression and in SGC7901/ADR and SGC7901/VCR cells with inhibited ITGA2 expression. **(D)** Protein levels of EMT biomarkers and apoptosis biomarkers in SGC7901 cells with ITGA2 overexpression and in SGC7901/ADR and SGC7901/VCR cells with inhibited ITGA2 expression.

To explore the underlying mechanism of ITGA2-induced GC chemoresistance, we performed gene expression profiling using the NanoString PanCancer Panel. NanoString analysis showed that ITGA2-silenced SGC7901/ADR cells displayed a different pathway expression profile than the control cells, and the MAPK pathway was distinguished as the one of the most downregulated pathways after ITGA2 knockdown (Figure 3A). Furthermore, pathway measurements identified positive correlations between the MAPK pathway and other pathways associated with drug resistance (Figure 3B). Next, we validated MAPK downstream kinases and found that MEK and ERK phosphorylation were significantly increased when ITGA2 was overexpressed and remarkably reduced after ITGA2 knockdown, whereas MDR1, an important protein of the cell membrane that pumps foreign substances out of cells, had no visible change (Figure 3C). The MAPK pathway also has profound effects on the regulation of apoptosis (35); we then tested the effects of ITGA2 on apoptotic regulatory molecules and found that ectopic expression of ITGA2 increased antiapoptotic protein Bcl-2 expression and decreased proapoptotic protein Bax expression. Conversely, knockdown of ITGA2 reduced Bcl-2 and increased Bax expression (Figure 3D). Accumulating evidence reveals that EMT contributes importantly to chemoresistance (36, 37). Since aberrant expression of integrins is involved in EMT, we speculate that ITGA2 mediates chemoresistance by affecting EMT in GC cells. We found that the epithelial marker E-cadherin was downregulated, while the mesenchymal marker Vimentin and N-cadherin were upregulated when ITGA2 was overexpressed. In contrast, silencing ITGA2 restored E-cadherin and suppressed Vimentin and N-cadherin expression (Figure 3D). Taken together, these results suggest that ITGA2 induces chemoresistance by activating the MAPK pathway and promotes EMT in GC cells.

### miR-135b-5p Directly Targets ITGA2 in GC Cells

**Figure 4:**
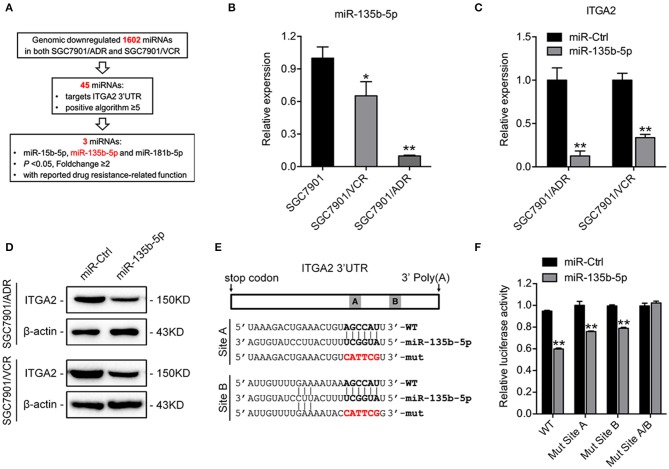
Mir-135b-5p inhibits ITGA2 expression by targeting its 3′-UTR. **(A)** Workflow for the identification of miR-135b-5p. **(B)** qRT-PCR analysis revealed that miR-135b-5p was downregulated in drug-resistant GC cells. **(C,D)** qRT-PCR analysis and Western blotting analysis showed that ITGA2 expression was inhibited when miR-135b-5p was overexpressed in drug-resistant GC cells. **(E)** Sequences of ITGA2 3′-UTR and miR-135b-5p. The binding sites miR-135b-5p and ITGA2 3′-UTR are shown in the bold portions. The mutant miR-135b-5p binding sites in the ITGA2 3′-UTR are shown in the red and bold portions. **(F)** Luciferase activity in 293T cells cotransfected with wild-type or mutated reporter plasmids, miR-ctrl, and miR-135b-5p. Data represent mean ± SD, from three replicates. *P < 0.05, **P < 0.01.

To investigate the regulatory mechanism of ITGA2 at the miRNA level, a bioinformatics strategy was employed to identify potential miRNAs targeting ITGA2 (Figure 4A). Briefly, we selected 1602 miRNAs that were downregulated in both SGC7901/ADR and SGC7901/VCR cells compared with that in SGC7901 cells according to our previous genomic profiling (38). Among them, 45 miRNAs were predicted to target the 3′-UTR of ITGA2 by using at least 5 different algorithms. We found that 3 out of 45 miRNAs (miR-15b-5p, miR-135b-5p, and miR-181b-5p) were significantly downregulated in chemoresistant cells (*P* < 0.05 and fold change>2) and were reported to be involved in drug resistance (39–41). We previously revealed the role and mechanism of miR-15b in GC multidrug resistance by targeting Bcl-2 (30). miR-181b-5p was excluded because its expression pattern contradicted the microarray data (Figure S1D). We thus focused on miR-135b-5p for further investigation and validated that its expression was reduced in both SGC7901/ADR and SGC7901/VCR cells, which was in contrast with the expression of ITGA2 (Figure 4B). Furthermore, qRT-PCR and Western blot analyses showed that overexpression of miR-135b-5p suppressed ITGA2 expression (Figures 4C,D, Figure S1E). To verify whether miR-135b-5p directly binds to the 3′-UTRs of ITGA2, we performed dual-luciferase reporter assays in 293T cells. miR-135b-5p overexpression suppressed the luciferase activities of the ITGA2 3′-UTR reporter wild-type constructs, whereas this effect was abolished when two mutations were introduced into the miR-135b-5p binding sequences in the mutant constructs (Figures 4E,F). Taken together, these results indicate that miR-135b-5p downregulates ITGA2 expression by directly targeting its 3′-UTR.

### miR-135b-5p Represses GC Cell Chemoresistance by Targeting ITGA2

**Figure 5:**
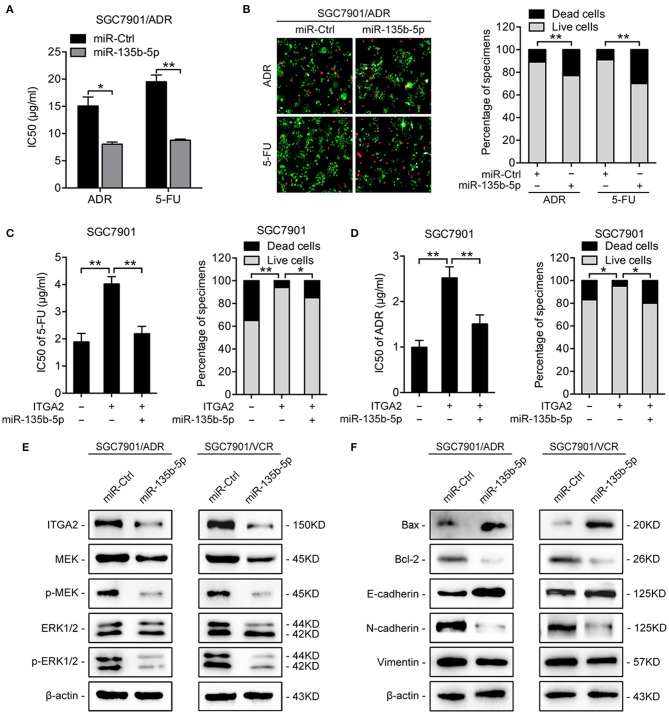
MiR-135b-5p overexpression-mediated chemoresistance. **(A)** IC50 of 5-FU and ADR when overexpressed miR-135b-5p in SGC7901/ADR cells. **(B)** LIVE/DEAD viability analysis in response to 5-FU and ADR when overexpressed miR-135b-5p in SGC7901/ADR cells. **(C,D)** IC50 and LIVE/DEAD viability analysis in response to 5-FU and ADR in SGC7901 cells infected with ITGA2 plasmids or vector and cotransfected ITGA2 plasmids and miR-135b-5p mimic. **(E)** Protein levels of the MAPK pathway markers were measured in SGC7901/ADR and SGC7901/VCR cells with miR-135b-5p overexpression. **(F)** Protein levels of apoptosis biomarkers and EMT biomarkers in SGC7901/ADR and SGC7901/VCR cells with miR-135b-5p overexpression. Data represent mean ± SD, from three replicates. *P < 0.05, **P < 0.01.

To investigate the functions of miR-135b-5p on GC chemoresistance, we transfected miR-135b-5p mimics into SGC7901/ADR and SGC7901/VCR cells. Upon miR-135b-5p overexpression, the IC50 of SGC7901/ADR cells to 5-FU and ADR was significantly decreased (Figure 5A). Consistently, LIVE/DEAD viability analysis showed that miR-135b-5p overexpression promoted apoptosis induced by 5-FU and ADR treatment (Figure 5B). Moreover, we cotransfected ITGA2-expressing plasmids with its 3′-UTR and miR-135b-5p into SGC7901 cells. As expected, the chemoresistance conferred by ITGA2 overexpression was partially antagonized by the ectopic expression of miR-135b-5p (Figures 5C,D, Figure S2A). In contrast to the effects of ITGA2, miR-135b-5p overexpression blocked MAPK signaling by decreasing the phosphorylation of MEK and ERK (Figure 5E). In addition, antiapoptotic Bcl-2 levels were reduced, whereas proapoptotic Bax levels were increased in miR-135b-5p-overexpressing cells (Figure 5F). Furthermore, miR-135b-5p also affected the characteristics of EMT in chemoresistant cells as the E-cadherin level increased, while Vimentin and N-cadherin expression decreased when miR-135b-5p was overexpressed (Figure 5F). Taken together, these results suggest that miR-135b-5p inhibits the chemoresistance of GC cells by regulating ITGA2 by blocking MAPK signaling and EMT.

## Discussion

Integrins trigger and play key roles in nearly all the malignant features that were described as the hallmarks of cancer (42, 43). Their roles in cell migration and invasion are one of the most studied functions in cancer biology. In head and neck cancer, knockdown of ITGA3 markedly suppressed cancer cell migration and invasion (44). In ovarian cancer, increased ITGB1 enhanced metastasis by mediating ECM remodeling (45). In GC, ITGAV overexpression was responsible for cell migration and invasion and was associated with poor prognosis (46). Recently, extensive studies have suggested that metastasis and drug resistance are intrinsically linked (47). For instance, EMT is a major mechanism of metastasis, but accumulating evidence indicates that EMT also contributes to chemoresistance (36, 48). Previous studies have underscored the importance of ITGA2 in metastasis. In liver cancer, upregulation of ITGA2 by ADAR1 enhanced metastasis by increasing adhesion to the ECM (16). In colorectal cancer, antibodies specifically blocking ITGA2 inhibited focal adhesion kinase activation and cell motility (18). However, the potential roles of ITGA2 in chemoresistance have not yet been determined. In the present study, we found that ITGA2 was increased in chemoresistant GC cells and high expression of ITGA2 correlated with poor prognosis of GC patients who received chemotherapy. Functional validation demonstrated that knockdown of ITGA2 restored the chemosensitivity of chemoresistant GC cells to 5-FU and ADR and that overexpression of ITGA2 induced chemoresistance to those drugs. Importantly, manipulation of ITGA2 triggered EMT marker alterations, as E-cadherin was dramatically reduced in GC cells overexpressing ITGA2, whereas silencing ITGA2 restored E-cadherin and repressed N-cadherin and Vimentin in the chemoresistant GC cells, implying that EMT and chemoresistance may intrinsically be linked via ITGA2.

Anticancer drug resistance can be divided into two categories: intrinsic resistance derived from genetic or environmental factors pre-existing in the tumor or acquired resistance resulting from adaptive responses, alternative pathway activation, and resistant subpopulation selection (10). Interaction between integrins and ECM contributes to both intrinsic and acquired resistance because it could be seen as a combined strategy that screens tumor cells with pre-existing integrin expression using a prosurvival and antiapoptotic method (49). MAPK signaling, leading to MEK/ERK activation, is a key regulator of cell proliferation and apoptosis and confers a survival advantage to cells (50). In leukemic Jurkat T cells, integrin α2β1 inhibited Fas-induced apoptosis though the collagen-mediated activation of the MAPK pathway (51). In breast cancer, ligation of integrin α3β1 with laminins protected tumor cells from anti-ErbB-2 agents via activation of the MAPK pathway (52). In the present study, we found ITGA2-mediated GC chemoresistance though the MAPK/ERK pathway. NanoString PanCancer Panel analysis identified that the MAPK pathway was one of the most deactivated signaling pathways and was associated with other drug resistance-related pathways after ITGA2 knockdown. The subsequent protein expression validation showed that ITGA2 affected the activation of the MAPK downstream kinase MEK and ERK. Activation of MAPK/ERK signaling has been shown to inhibit apoptosis. Consistently, we observed that ITGA2 influenced the apoptotic regulatory molecules Bcl-2/Bax in GC cells. However, it is noteworthy that other molecules or pathways may also contribute to the effect of ITGA2 on GC chemoresistance, which remains to be further investigated.

miRNAs are important regulators of gene expression and are frequently dysregulated in cancer. A single miRNA could affect the translation of multiple genes and may lead to profound phenotypic responses within a cell (53). Aberrant expression of miR-135b has been reported in various cancers and has been shown to regulate multiple malignant phenotypes. In pancreatic cancer, miR-135b-5p inhibited cell migration and invasion by regulating NR3C2 (54). In breast cancer, overexpression of miR-135b-5p facilitated apoptosis and reduced chemoresistance to doxorubicin by targeting pro-oncogenic AGR2 (41). In non-small cell lung cancer, amplification of miR-135b suppressed the chemoresistance of cancer cells to cisplatin treatment by downregulating Frizzled-1 (55). However, opposite effects of miR-135b-5p in certain cancers have also been reported (56, 57). This could be partially explained by the mechanism by which miRNA binding to mRNAs is often achieved with imperfect complementarity, and its targets varied in different cellular contexts and tumor types. In the present study, we provided several lines of evidence that support miR-135b-5p function as a tumor suppressor of chemoresistance by targeting ITGA2 in GC. First, miR-135b-5p is reduced in chemoresistant GC cells, and its overexpression induced apoptosis and restored the sensitivity of chemoresistant GC cells to chemotherapeutic drugs. Second, dual-luciferase reporter assays confirmed the direct interaction between miR-135b-5p and ITGA2, and the chemoresistance conferred by ITGA2 overexpression was partially antagonized by miR-135b-5p ectopic expression. Third, miR-135b-5p also influences the MAPK/ERK pathway, apoptotic regulatory molecules and EMT-related markers. These observations suggest that miR-135b-5b might be a potential therapeutic agent to target ITGA2 for GC chemoresistance treatment.

In summary, our study investigated the potential role and mechanisms of ITGA2 in GC chemoresistance. Based on our findings, the ability of ITGA2 to control the MAPK pathway and EMT may lead to GC chemoresistance. Our findings revealed a novel miR-135b-5p/ITGA2 axis, implying that this axis may represent a new mechanism of chemoresistance and that this axis holds promise for the development of potential therapeutics against GC.

## Data Availability Statement

All datasets generated for this study are included in the article/Supplementary Material.

## Ethics Statement

All animal studies were approved by the Fourth Military Medical University Animal Care Committee and conducted according to the Association for Assessment and Accreditation of Laboratory Animal Care and the Institutional Animal Care and Use Committee guidelines.

## Author Contributions

XZ, YL, and DF designed the experiments. QW, TC, and YZ performed the experiments. QW and TC drafted the manuscript. QW, HL, KG, QH, YP, and YN conducted the data analysis and interpreted the results. XZ and YL revised the manuscript. All the authors reviewed and approved the manuscript.

### Conflict of Interest

The authors declare that the research was conducted in the absence of any commercial or financial relationships that could be construed as a potential conflict of interest.

## Supplementary Material

Figure S1 The effect of ITGA2 on drug-induced apoptosis and the expression of miR-181b-5p and miR-135b-3p. **(A)** Western blotting analysis and qRT-PCR analysis of ITGA2 expression in when silencing or overexpressing ITGA2 in SGC7901, SGC7901/VCR, and SGC7901/ADR cells. **(B)** LIVE/DEAD viability analysis in response to ADR when inhibited ITGA2 expression in SGC7901/ADR cells. **(C)** IHC of Ki-67 staining and Cleaved Caspase-3 staining in nude mice tissues upon 5-FU or ADR treatment. **(D)** qRT-PCR analysis of miR-181b-5p expression in SGC7901, SGC7901/VCR, and SGC7901/ADR cells. **(E)** Transfection efficiency of miR-135b-5p mimic in SGC7901/ADR and SGC7901/VCR cells.

Figure S2 miR-135b-5p reversed ITGA2 overexpression induced chemoresistance. A LIVE/DEAD viability analysis in response to 5-FU and ADR in SGC7901 cells infected with ITGA2 plasmids or vector and cotransfected ITGA2 plasmids and miR-135b-5p mimic.

Figure S3 Original images of western blots. **(A)** Original images of Figure 1A, **(B)** Figure 3C, **(C)** Figure 3D, **(D)** Figure 4D, **(E,F)** Figures 5E,F, **(G)** Figure S1A.
