# Supplementary figures and images for: Regulation of Integrin Subunit Alpha 2 by miR-135b-5p Modulates Chemoresistance in Gastric Cancer
Source: Front Oncol. 2020 Mar 13;10:308. doi: 10.3389/fonc.2020.00308 (PMC7082357; doi:10.3389/fonc.2020.00308)

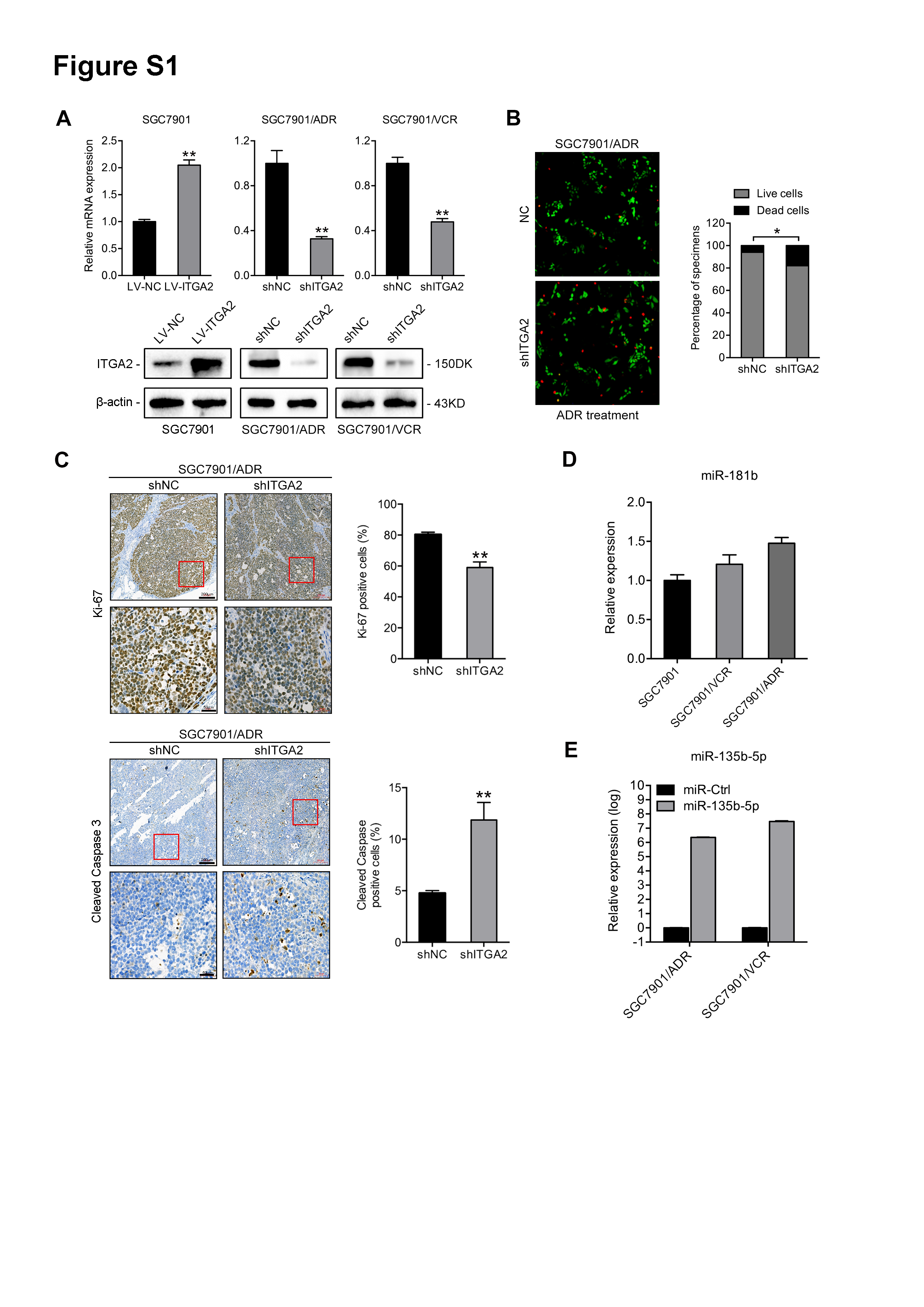

Supplement: Figure S1 — The effect of ITGA2 on drug-induced apoptosis and the expression of miR-181b-5p and miR-135b-3p. (A) Western blotting analysis and qRT-PCR analysis of ITGA2 expression in when silencing or overexpressing ITGA2 in SGC7901, SGC7901/VCR, and SGC7901/ADR cells. (B) LIVE/DEAD viability analysis in response to ADR when inhibited ITGA2 expression in SGC7901/ADR cells. (C) IHC of Ki-67 staining and Cleaved Caspase-3 staining in nude mice tissues upon 5-FU or ADR treatment. (D) qRT-PCR analysis of miR-181b-5p expression in SGC7901, SGC7901/VCR, and SGC7901/ADR cells. (E) Transfection efficiency of miR-135b-5p mimic in SGC7901/ADR and SGC7901/VCR cells. [file Image_1.TIF]

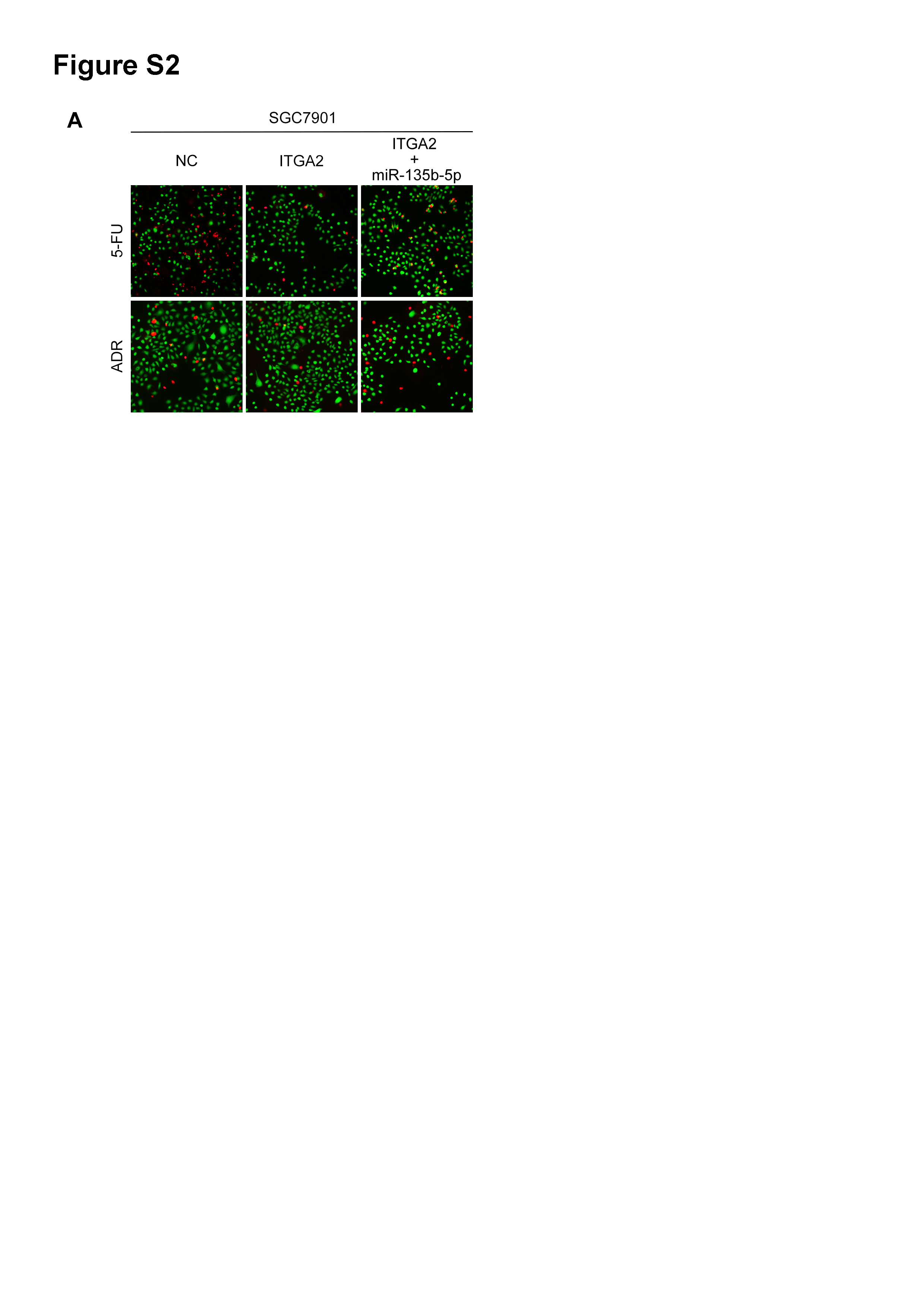

Supplement: Figure S2 — miR-135b-5p reversed ITGA2 overexpression induced chemoresistance. A LIVE/DEAD viability analysis in response to 5-FU and ADR in SGC7901 cells infected with ITGA2 plasmids or vector and cotransfected ITGA2 plasmids and miR-135b-5p mimic. [file Image_2.TIF]

Figure S3

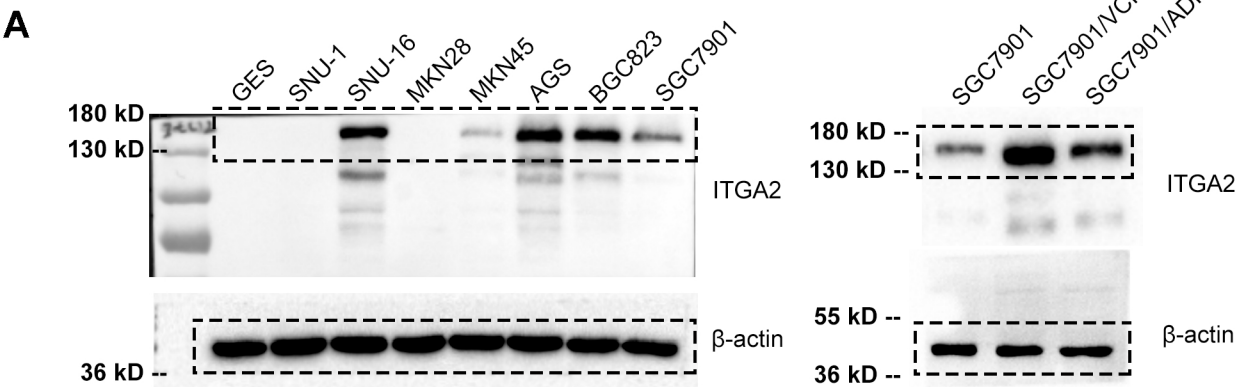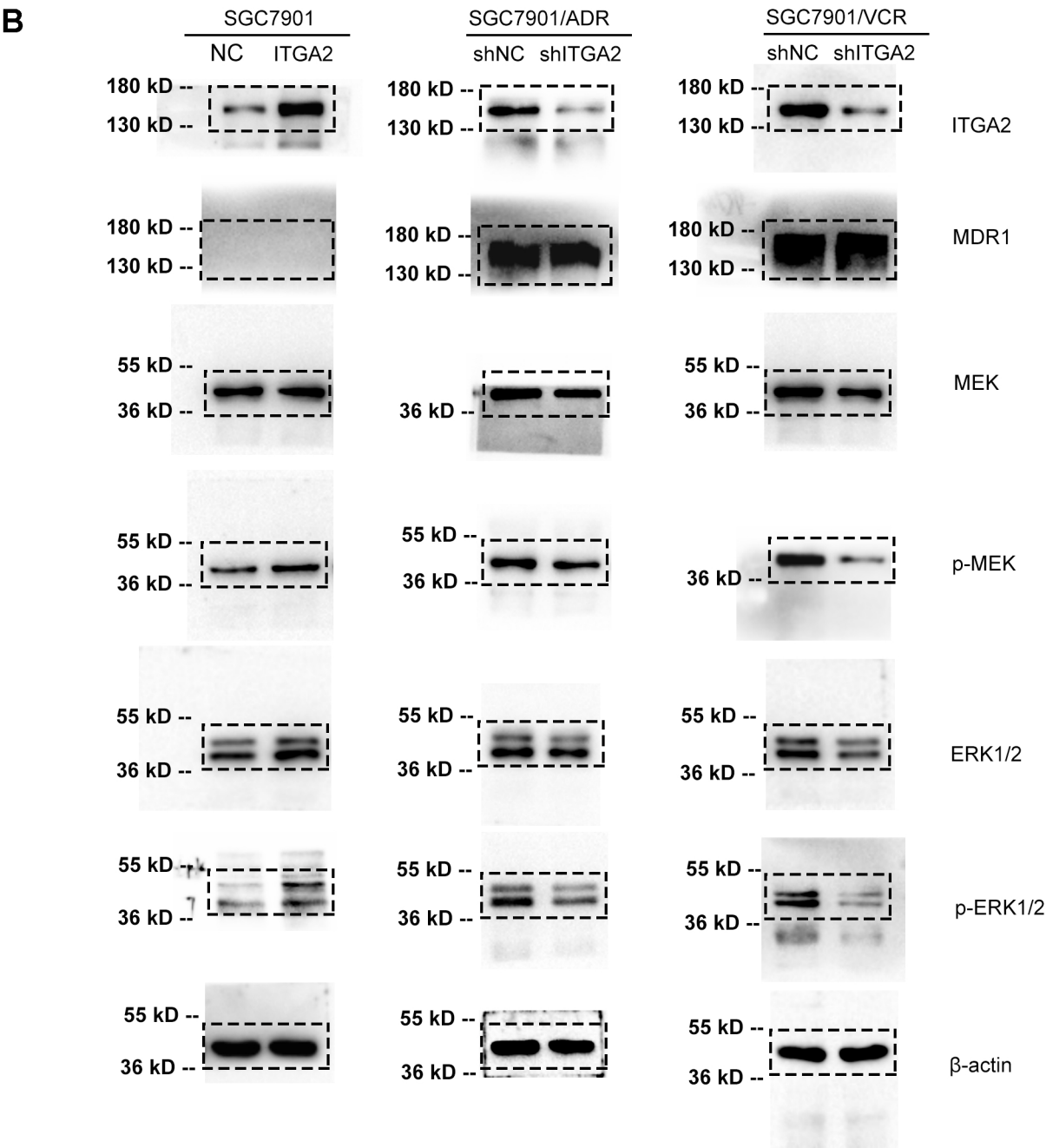

**C**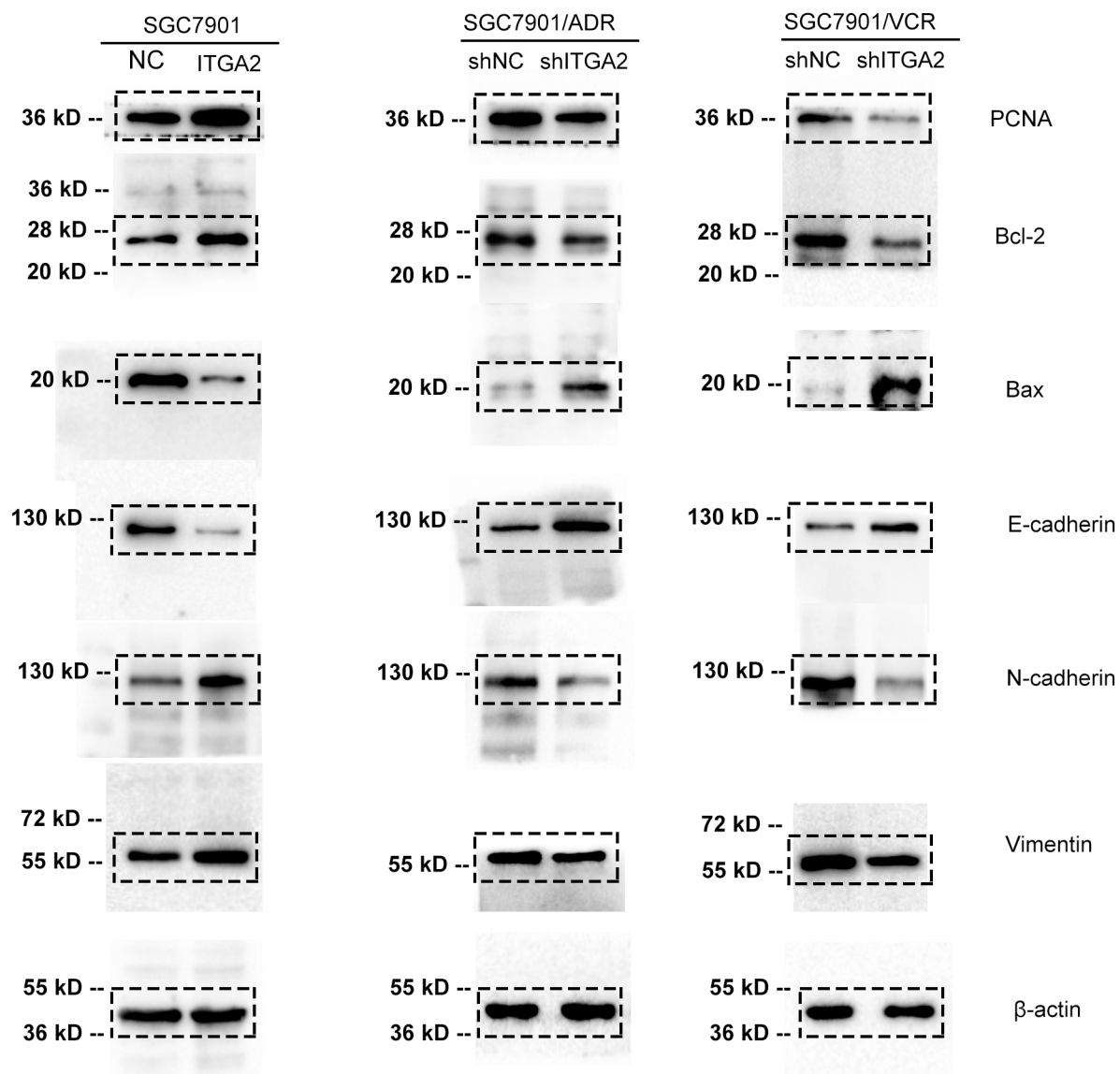**D**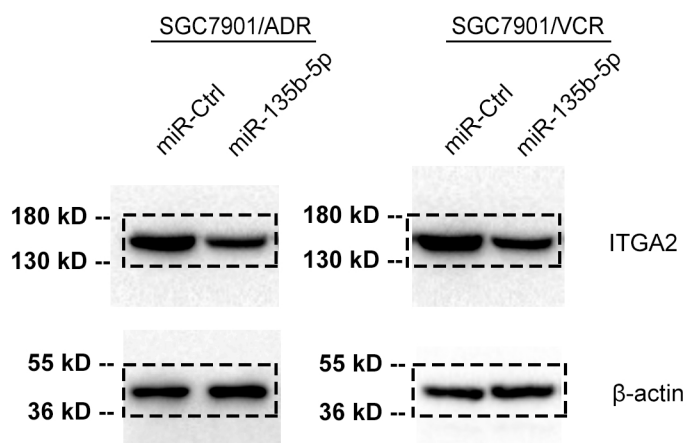

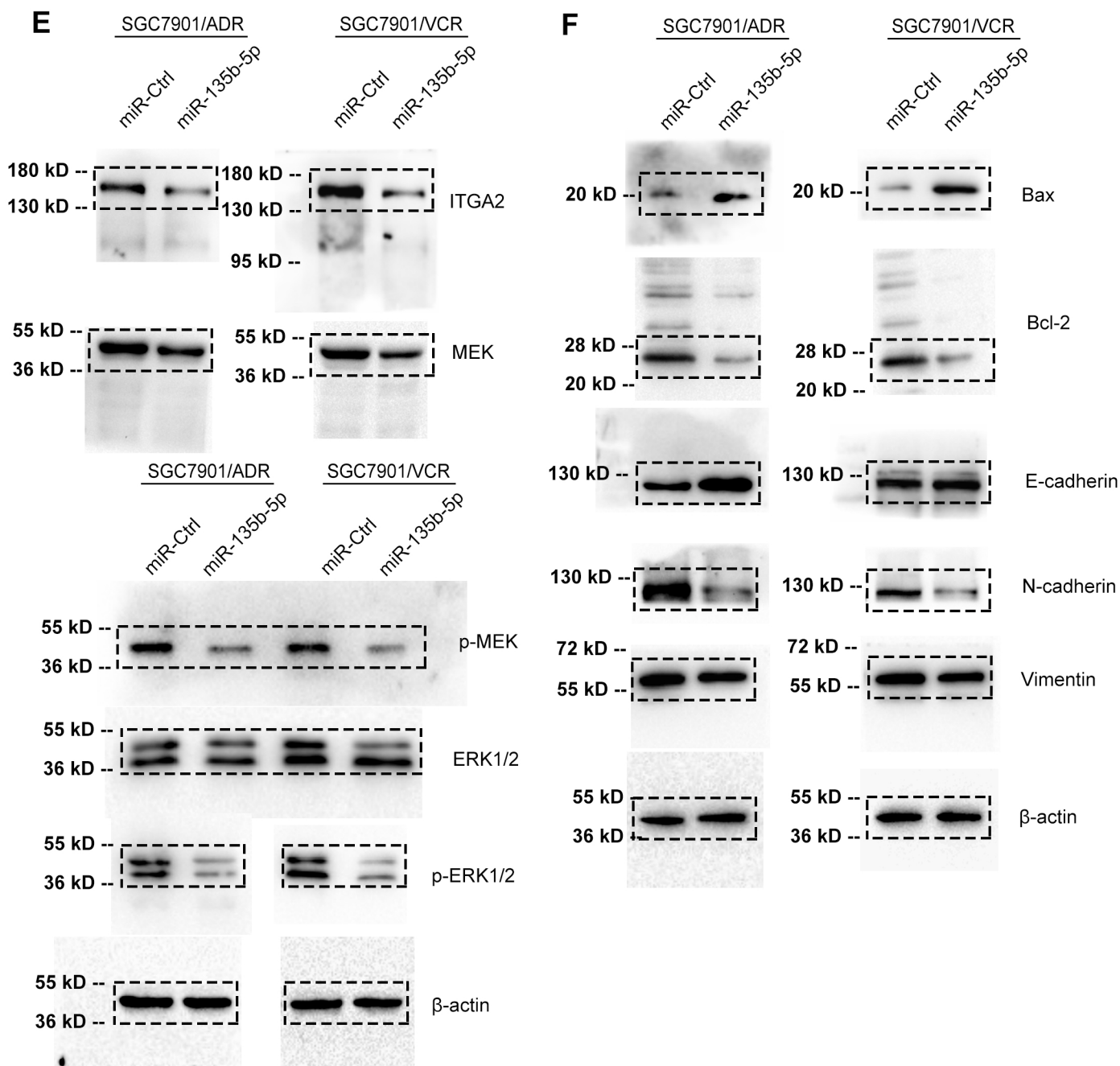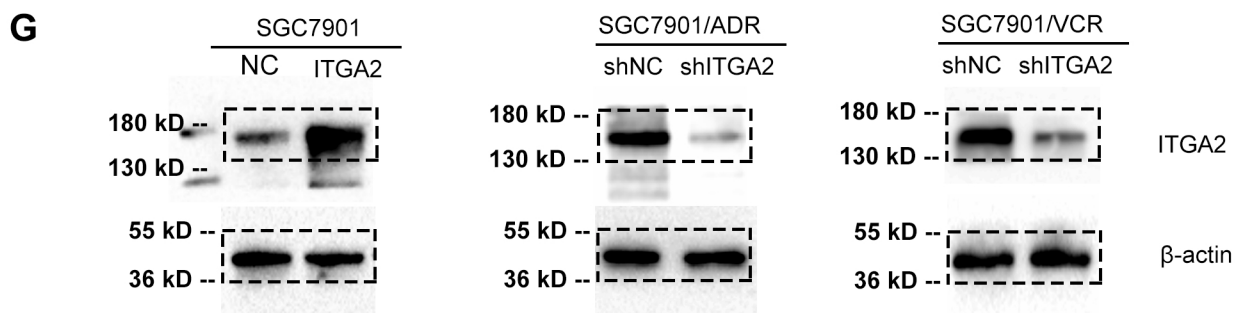

Supplement: Figure S3 — Original images of western blots. (A) Original images of Figure 1A, (B) Figure 3C, (C) Figure 3D, (D) Figure 4D, (E,F) Figures 5E,F, (G) Figure S1A. [file Image_3.pdf]
